# Biomedical Application of Photoacoustics: A Plethora of Opportunities

**DOI:** 10.3390/mi13111900

**Published:** 2022-11-03

**Authors:** Deblina Biswas, Swarup Roy, Srivathsan Vasudevan

**Affiliations:** 1 School of Bioengineering and Food Technology, Shoolini University, Solan 173229, HP, India; 2 Discipline of Electrical Engineering, Indian Institute of Technology Indore, Khandwa Road, Simrol 453552, MP, India

**Keywords:** photoacoustics, tomography, microscopy, signal processing, biomedical applications

## Abstract

The photoacoustic (PA) technique is a non-invasive, non-ionizing hybrid technique that exploits laser irradiation for sample excitation and acquires an ultrasound signal generated due to thermoelastic expansion of the sample. Being a hybrid technique, PA possesses the inherent advantages of conventional optical (high resolution) and ultrasonic (high depth of penetration in biological tissue) techniques and eliminates some of the major limitations of these conventional techniques. Hence, PA has been employed for different biomedical applications. In this review, we first discuss the basic physics of PA. Then, we discuss different aspects of PA techniques, which includes PA imaging and also PA frequency spectral analysis. The theory of PA signal generation, detection and analysis is also detailed in this work. Later, we also discuss the major biomedical application area of PA technique.

## 1. Introduction

Photoacoustic (PA) or optoacoustic technique is an undoubtedly appealing image- and signal-based diagnostic technique this decade which has attracted the attention of scientists from different domains, as well as clinicians. As the name suggests, PA technique is a unique blend of two different fields: photo (optics) and acoustics (sound). The features that make this technique most popular among researchers are that it is non-invasive, non-ionising, has high optical absorbance contrast and sub-millimeter resolution deep (~5 cm) inside the tissues and organs, which is desirable for advanced diagnosis [1,2]. Unlike ultrasound, PA imaging is based on optical absorbance of the targeted tissue chromophores (called contrast agents), such as haemoglobin, lipid, water, melanin, etc., which thereby enhances the specificity of the technique [3,4]. The sample is probed with very short laser pulses (nano-second laser pulses) that generate non radiative, broad band acoustic signals, due to thermoelastic expansion and compression of the sample. These acoustic signals are bipolar in nature, known as PA response. Subsequent to acquisition, the PA responses are fed into reconstruction algorithms (e.g., time reversal, back projection) to form an image [5,6,7]. The primary advantage of this technique is that it relies on detection of acoustic waves rather than photons (the same as a purely optical technique), as acoustic waves are less prone to scattering, as well as attenuation compared to light. As a result, PA imaging yields higher spatial distribution of optical absorbance contrast deep inside the tissue that compliments the existing purely optical techniques. Not only does the technique have high optical absorption contrast and penetration depth, PA imaging (PAI) elucidates a spatial resolution of ~10 μm, which is ascendable with the frequency of an ultrasonic sensor, similar to ultrasound imaging [8,9]. In addition to PA imaging, PA time domain frequency spectral analysis also provides critical information on physical, biochemical and mechanobiological details about biological tissue [10,11]. Due to these unique features of the PA technique, it has been employed to study different diseases, including cancer [12,13,14,15]. In this review, we discussed different types of PA techniques and various biomedical applications in recent times. Among the PA techniques, PAI is the most popular diagnostic modality. However, the PA time domain signal carries critical information about biological tissue pathology which can be extracted by applying different signal processing tools to the PA time domain signal. Even though PA signal analysis possesses enormous diagnostic potential, there are very few review articles that detail this potential. Moreover, articles which explain the diagnostic features of PA techniques (imaging and signal processing) are very rare. Thus, this article details different PA imaging techniques, PA signal pre-processing techniques, different PA signal analysis techniques and their biomedical applications, collectively.

## 2. About PA Technique

### 2.1. PA Wave Generation and Propagation

The basic physics behind the photoacoustic technique was first observed by Alexander Graham Bell in 1880, during his experiment creating the photophone [16]. He observed that when a selenium cell was illuminated with modulated light, sound waves were generated. Since incidence of light caused sound generation, the physical phenomenon was named as the photoacoustic effect.

In order to generate sound using light, the primary requirements are given as follows [3]:

- The sample should absorb the irradiated energy in terms of light or electromagnetic waves
- The energy should be in modulated form

If the above-mentioned conditions are fulfilled, then the PA phenomenon can be observed. Usually for PA wave generation, short laser pulses are incident on the sample surface. The light penetrates through the sample depending upon the wavelength. Subsequently, the light gets scattered and absorbed by the lattice specific molecules of the sample. PA works on the principle of short excitation which is shorter than two important time frames which are as follows [3,17]:

- *Thermal relaxation time* is basically estimated by the thermal diffusion shown in Equation (1).
  $$\tau_{th}=\frac{d_{c}^{2}}{\alpha_{th}}$$
  where *α_th_* is thermal diffusivity (m^2^/s) and *d_c_* is the linear characteristic length of the heated regime of the sample.
- The *stress relaxation time* is associated with pressure propagation, i.e., the laser pulse duration should be less compared to the time taken for release of stress from the heated regime. This is expressed as
  (2) $$\tau_{s}=\frac{d_{c}^{}}{V_{s}}$$
  where *V_s_* is speed of sound.

Now, if the laser pulse width is much smaller than the thermal relaxation time, then a thermal confinement condition arises. If thermal diffusivity is considered to be 0.14 mm^2^/s, then for imaging a target with 100 μm resolution, it would require an 18 ns laser pulse according to Equation (1). Consequently, for stress confinement, the laser pulse width should be short compared to stress relaxation time. This is required for thermoelastic expansion of the sample which thereby causes acoustic signal generation. Similarly, to achieve 100 μm spatial resolution in soft tissue imaging (sound speed ~1500 m/s), the laser pulse width should be 60 ns according to Equation (2). Thus, it is clear from the above explanation that the laser pulse width should be in nano second duration in order to achieve submillimeter resolution. Fulfilment of these basic criteria initiates PA wave generation by enhancing the temperature of the sample followed by fractional volume change which is given as [3]

(3) $$\frac{dV}{V}=-\kappa p+\beta T$$

where *κ* is isothermal compressibility which is ~5 × 10^−10^ Pa^−1^ for water and soft tissue, *β* represents thermal coefficient of volume expansion (~4 × 10^−4^ K^−1^ for muscle) [3], *p* and *T* represent the change in pressure and temperature.

The isothermal compressibility is expressed as [1]

(4) $$\kappa =\frac{C_{p}}{dV_{s}^{2}C_{V}}$$

here *d* is mass density and *C_p_*, *C_V_* are the specific heat constants at constant pressure and volume, respectively. If both the criteria of thermal and stress confinements are fulfilled then there is a negligible amount of volume expansion, i.e., [3]. The thermal expansion of the sample is followed by enhancement in initial pressure expressed as [1]

(5) $$\Delta p_{0}=\frac{\beta \Delta T}{K}=\frac{\beta }{\kappa dC_{V}}\eta_{th}A_{e}$$

Here, *A_e_* is denoted as optical absorption (J/m^3^) and *η_th_* is the percentage that is converted into heat. This can be represented as

(6) $$\Delta p_{0}=\Gamma A_{e}$$

(7) $$\Gamma =\frac{\beta }{KdC_{V}}=\frac{\beta V_{s}^{2}}{C_{p}}$$

The dimensionless parameter is named as the Gruneisen coefficient, that consists of a thermal expansion coefficient, compressibility parameters and sound speed. The equation can be rewritten as

(8) $$\Delta p_{0}=\Gamma \eta_{th}A_{e}=\Gamma \eta_{th}\mu_{a}F$$

Here, *μ_a_* is optical absorption coefficient and *F* is fluence.

It can be clearly observed that a short laser pulse satisfies the confinement condition as well as ensures temperature rise to initial pressure increase when the Gruneisen coefficient is maximum. This initial pressure acts as a source of PA waves, which depend on laser energy absorption and scattering property, thermal properties such as thermal diffusivity and thermal expansion coefficient as well as the elastic property of the material.

The governing equation of photoacoustic wave generation and propagation in an acoustically homogeneous medium is given as follows [18]:

(9) $$\left(\nabla^{2}-\frac{1}{V_{s}^{2}}\frac{\partial^{2}}{\partial t^{2}}\right)p(r,t)=-\frac{\beta }{\kappa V_{s}^{2}}\frac{\partial^{2}T(r,t)}{\partial t^{2}}$$

where *p(r,t)* represents acoustic pressure at location *r* and time *t*, *T* indicates the rise in temperature.

The source term is detailed in the right side and wave propagation is illustrated in the left part of the wave equation.

The generated PA waves are three-dimensional (spherical) and a time-dependent signal which propagates through the sample in longitudinal mode (compression and rare fraction). Since PA waves are longitudinal waves, it is well associated with the particle velocity, density and the sound speed of the sample, as well as the propagation medium that is given as [19,20].

(10) P(x,y,z,t)=v(x,y,z,t)×ρ×c

PA waves elucidate basic properties such as reflection and refraction at the boundaries of different density, which follows Snell’s law, expressed as follows [20]:

(11) $$\frac{\sin\theta_{i}}{V_{s1}}=\frac{\sin\theta_{r}}{V_{s1}}=\frac{\sin\theta_{t}}{V_{s2}}$$

where *θ_i_*, *θ_r_*, *θ_t_* are the angle of incidence, reflection and transmission with respect to the axis and *V*_*s*1_, *V*_*s*2_ is the sound speed in the two mediums.

During propagation, generated PA waves are also affected by attenuation which is combined contribution of scattering and absorption of acoustic wave in the sample and the medium. The attenuation depends on the temperature and frequency that can be expressed as [21,22,23]:

(12) $$A^{\prime }=A_{0}e^{-a\prime z}$$

where, *A*_0_ is amplitude attenuation factor (dB/MHz^−1^ cm^−1^), *z* is distance.

In case of biological tissues, usually the acoustic attenuation is very low compared to optical attenuation. The acoustic attenuation depends on the tissue type as well as the frequency of the traversing acoustic wave. However high frequency acoustic waves are more susceptible to attenuation. As a result, the acoustic signal would be very weak or die out before reaching the sensor. This will lead to decrease in penetration depth.

#### Laser Safety Standard

In order to ensure the safety of the subjects, a certain limit of radiation exposure was decided by American National Standard Institute (ANSI). The limit depends on the laser wavelength, pulse duration, exposure duration and exposure aperture. The ANSI requirement policy states that “Exposure of the skin shall not exceed the MPE based upon a single-pulse exposure, and the average irradiance of the pulse train shall not exceed the MPE applicable for the total pulse train, duration T” (Laser Institute of America 2000). For example, if a pulsed laser source generates a second harmonic laser pulse (532 nm) with 5 ns pulse width, which irradiates the skin at the same region in area of 1 cm^2^ for more than 10 s, then the safety standard for one single pulse would be 20 mJ (less than MPE). In addition, the average power also should be less compared to MPE, i.e., 200 mW. Basically, long exposure is required for photoacoustic microscopy which needs raster scanning. In that case, the safety limit can be expressed as follows [24]:

(13) E×Fr4≤2.75×102πda54(Δ/Ni)34

where *E* is the pulse energy in mJ, *F_r_* is the repetition rate in Hz, *d_a_* represents the illumination spot diameter in cm, Δ denotes the scanning step size in cm and *N_i_* is the number of pulses at each spot.

### 2.2. PA Signal Detection

**Figure 1:**
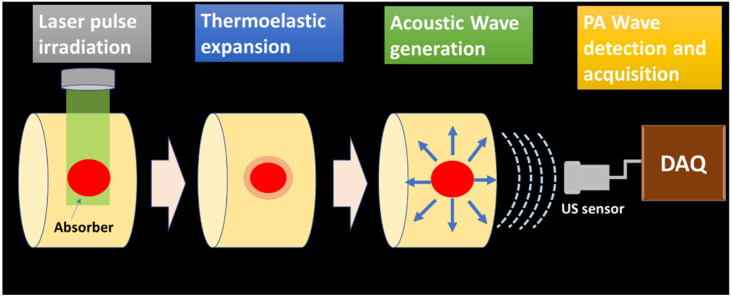
PA wave generation and propagation schematic.

The generated PA waves are propagated through the sample and the coupling medium (water, ultrasonic gel for biological tissue imaging). These signals are acquired by the ultrasonic sensor placed around the sample surface. The schematic of PA wave generation and propagation is illustrated in Figure 1.

#### Ultrasound Sensors

For PA applications, commonly used ultrasonic sensors are made of lead zirconate titanate (PZT) which is a piezoelectric crystal [25]. The basic principle of piezoelectric crystal relies on piezoelectric effect, i.e., when potential difference is applied to opposite sides of the crystal surface, mechanical displacement is observed. Similarly, application of mechanical force produces potential difference. Since acoustic waves can generate mechanical force, piezoelectric crystals provide voltage difference which is proportional to acoustic wave intensity [26]. The piezoelectric crystal is the primary component of the ultrasonic sensor which is placed at the forefront of the sensor. The bandwidth and centre frequency of the sensor is controlled by the thickness of the crystal. In order to ensure electrical conduction, the crystal is coated with conducting material. Subsequently, electrodes are placed at the front and back side of the crystal. This is followed by the backing material that provides damping to the sensor by absorbing the reflected acoustic signal. The sensor contains an acoustic insulator to prevent detection of external acoustic wave and internally generated acoustic wave in the crystal [27,28]. The entire arrangement is placed inside insulated casing. The PA signals are sensed by the crystal, which converts pressure waves to electrical signal. Even though, piezo-based US sensors are the most common sensor PA signal acquisition, they conventionally possess a narrow bandwidth. The wideband piezo sensors are fabricated of thin films which are not robust. Therefore, optical acoustic sensors are explored for PA signal acquisition as they are inherently wide bandwidth, can be easily miniaturised and have high sensitivity. Primarily, refractometry and interferometry methods are employed for US detection [27]. In case of the refractometric US sensor, an acoustic pressure wave induces change in the refractive index of the wave propagating medium. This change in refractive index is proportional to acoustic pressure. Intensity-sensitive detection of the refractive index, single-beam deflectometry, is an example of a refractometric US sensor. On the other hand, change in the optical interference pattern by acoustic pressure is the underlying principle for the interferometric US sensor. Phase detection in a Michelson interferometer, and Doppler-based sensing are examples of interferometric US sensors [27,28].

### 2.3. Characteristics of Time Domain PA Signal

**Figure 2:**
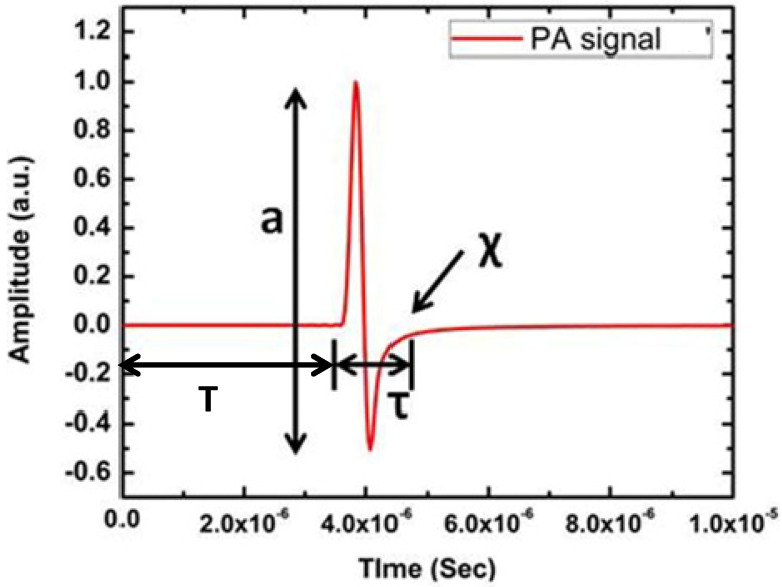
A typical PA signal from circular numerical target where a is amplitude, T is time of flight, τ is width and χ is the relaxation time of the PA time domain signal.

Since PA waves are longitudinal waves (soft tissues, water), it consists of compression and rarefaction. Thus, the piezoelectric ultrasonic sensor produces a bipolar signal that has a resemblance to the letter “N”. A typical PA signal from a circular simulated target is illustrated in Figure 2.

Figure 2 clearly shows the primary features of the PA time domain signal. It consists of four dominating features, namely, amplitude (a), delay (T), width (τ) and relaxation time (χ). Among these features, width and relaxation time are related to each other. These features of PA time domain signal indicate very important properties of the sample. For example, PA signal amplitude is related to a sample’s optical absorption and laser energy irradiated onto the sample, delay provides information about the position of the absorber, width of the signal represents the size of the absorber and relaxation time depicts the elastic property of the sample [29,30]. Therefore, PA signal consists of critical information about the sample. For image reconstruction, only two features such as amplitude and delay are utilised. Signal amplitude appears as contrast in the image and time delay provides the depth information of the absorber. Since normal and pathological tissues exhibit distinct change in optical absorption, PA imaging provides high contrast among these tissues [31,32].

### 2.4. PA Image Reconstruction

#### 2.4.1. PA Signal Pre-Processing

The PA time domain signal acquired by the ultrasonic sensor is highly affected by the noise as well as the bandwidth of the sensor, which alters the actual profile of the PA signal. Hence, denoising and deconvolution with sensor response is essential to improve the signal-to-noise ratio (SNR) of the reconstructed image, since the PA image contrast depends on the amplitude of the PA signal [24,33,34].

The PA signal denoising can be performed by techniques including averaging, filtering, wavelet-based denoising, etc. [35,36,37]. The random noise is usually eliminated by performing time-averaging of the signal by conducting multiple acquisitions from the same point. However, averaging is affected by different artefacts such as a patient’s movement, heartbeat, etc. Therefore, different techniques, such as moving time averaging and frequency filtering, can be performed to remove the noise [24]. The moving averaging method is substantially used for eliminating high frequency noise when the signal is in a low frequency regime, whereas frequency filtering is applied to the signal with minimum overlap in noise and signal. Since the presence of multiple targets broadens the PA frequency spectrum, the application of moving averaging removes the high frequency component and frequency filtering eliminates the useful frequency components overlapped with noise. Hence, wavelet-based denoising has gained a lot of attention. In contrast to Fourier transformation and other signal processing techniques, wavelet decomposes the time domain signal into a scalable window function with different coefficient values [38,39,40,41]. A mother wavelet function is used for deriving wavelet window functions by performing transformation and scaling. Thus, wavelet transform is applied to the PA time domain signal, which removes the noise. Subsequently, the inverse wavelet transform is performed to recover the denoised signal.

The other important factor which affects the PA signal characteristics is the limited bandwidth of the sensor. This may distort the actual profile of the PA time domain signal, as the acquired PA time domain signal is the convolution of generated acoustic pressure and sensor’s impulse response expressed as [24].

(14) $$p_{d}(r,t)=p(r,t)*d_{\delta }(t)$$

where *d_δ_* is the sensor’s impulse response

In frequency domain initial pressure is expressed as

(15) $$p(r,\omega )=\frac{p_{d}(r,\omega )}{d_{\delta }(\omega )}$$

In order to recover the original pressure profile, inverse Fourier transform can be performed. However, this will magnify the noise manyfold. Thus, the PA signal is deconvoluted by performing zero routine and Wiener deconvolution. The following equations illustrate both zero routine and Wiener deconvolution [42]:

(16) $$p(r,\omega )=\left[\frac{d_{\delta }^{*}(\omega )|p_{d}(r,\omega ){|}^{2}}{|d_{\delta }(\omega ){|}^{2}|p_{d}(r,\omega ){|}^{2}+\sigma_{n}^{2}}\right]p_{d}(r,\omega )$$

(17) $$p(r,\omega )=\frac{d_{\delta }(\omega )p_{d}(r,\omega )}{d_{\delta }{\left(\omega \right)}^{2}+\delta^{2}}$$

#### 2.4.2. Image Reconstruction Algorithm

The denoising process of the acquired PA signal is followed by image reconstruction. The processed PA signals are fed into different reconstruction algorithms such as back projection, time reversal, fast Fourier transform algorithm, etc. for image reconstruction [1,43,44]. The image quality, as well as imaging speed, depends on the reconstruction algorithm.

In the case of back projection, the sum of initial pressure is detected by each element. Each acquisition point provides information about the point source as well as total attenuation along the path. The acquired information is projected back to reconstruct the image [45].

Advancement in the reconstruction algorithm provides more accurate reconstruction and better computational efficiency. For example, the back projection algorithm is modified to filtered back projection, which employs filtering before or after the back projection step. This reduces the artefacts in the reconstructed image. While this algorithm is computationally efficient, it is limited by the spherical geometry in practical application [3,46].

In contrast, the time reversal algorithm utilises the temporally reversed PA waveform collected from each detection point for image reconstruction [7]. Briefly, PA signals acquired by each sensing point are temporally reversed and retransmitted in the medium numerically to trace the origin of that particular point source. The primary advantage of this algorithm is that it relies on the least number of assumptions and is applicable for any geometry [47,48]. However, the time reversal algorithm demands huge memory for computation that limits the practical application of this algorithm. The memory requirement of the conventional time reversal technique is reduced by an efficient technique called the pseudo-spectral K-wave propagation model [49,50]. This employs a pre-computation of initial pressure by a forward model. Then matrix-based and simulated p_0_ would reduce the memory requirement for computation.

## 3. Types of PA Technique

In the biomedical field, PA technique is mainly utilised for imaging applications. However, this has been also explored for spectroscopy (gas and liquid analysis) and application to PA signal analysis.

**Figure 3:**
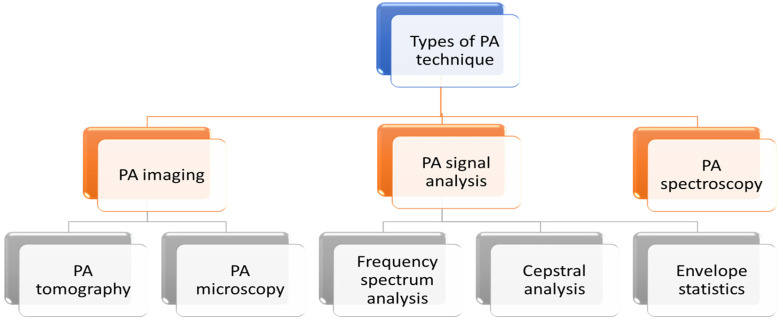
Different types of PA techniques.

PA technique can be categorised in three broad applications: imaging, spectroscopy and PA signal analysis. PA imaging is further divided into two types: PA tomography and PA microscopy. Different types of PA techniques are detailed in the following flowchart (Figure 3).

### 3.1. PA Imaging

#### 3.1.1. Photoacoustic Tomography (PAT)

**Figure 4:**
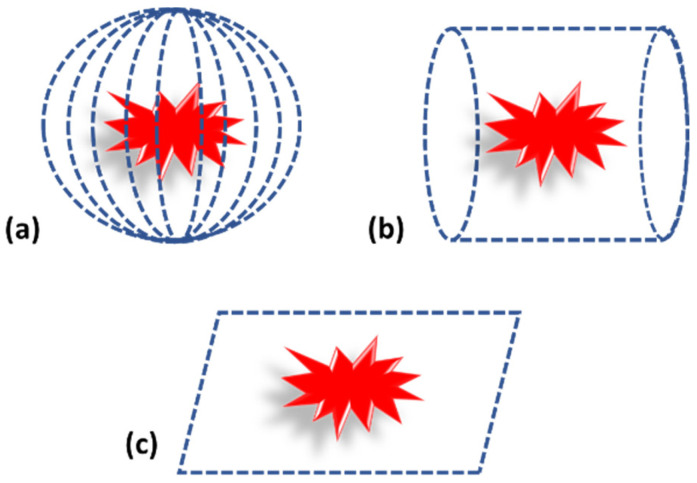
Photoacoustic tomography detection geometry. (**a**) Spherical. (**b**) Cylindrical. (**c**) Planar.

Photoacoustic tomography can be considered as the traditional way of performing imaging. In case of photoacoustic tomography, a single transducer is moved around the sample in different geometric positions, such as planar, circular and spherical, to acquire the PA pressure waves, as shown in Figure 4 [1,43]. The scanning time with a single element sensor is quite high. Therefore, an array of sensors is also used for PA signal acquisition [20,51]. Since the cost of the array sensor is relatively high compared to a single element sensor, these are used in a limited manner.

While considering the detection geometry, it mainly depends on the target tissue. For example, for brain or breast imaging, spherical and cylindrical geometry is used, but for flat targets such as bone or skin, planar geometry is used [2,5,43,52]. Subsequent to acquisition, PA signals are fed into suitable reconstruction algorithms for image reconstruction. Details of reconstruction algorithms are explained in earlier section.

#### 3.1.2. Photoacoustic Microscopy (PAM)

In contrast to PAT, photoacoustic microscopy does not require any robust image reconstruction algorithm to form an image. It utilizes a tightly focused laser beam or focused ultrasonic detector to form an image. In the case of PAM, either the focused laser beam or focused acoustic detector is mechanically scanned through the sample. PA signals obtained from individual detection points directly form an image without aid of any reconstruction algorithm [9,53].

Depending upon the focused laser spot size on the sample, PAM can be categorised as acoustic resolution PAM (AR-PAM) and optical resolution PA microscopy (OR-PAM). The schematics of these two microscopy techniques is illustrated as follows [43,54,55].

AR-PAM utilises a focused ultrasound detector whereas OR-PAM relies on a focused laser beam for scanning. The axial resolution of the PAM depends upon the time-resolved detection of the PA signal but the lateral resolution relies on either acoustic focusing or optical focusing [33].

### 3.2. PA Spectroscopy

**Figure 5:**
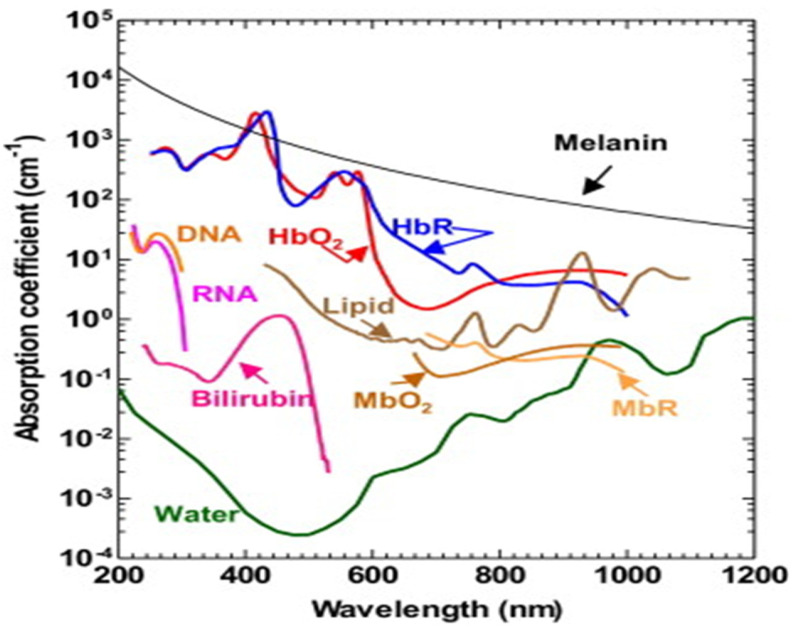
Optical absorption spectra of different tissue chromophores [9].

The PA time domain signal consists of much critical information about the sample. As mentioned in the previous section, it substantiates optical absorption, size, mechanobiological property of the sample by its amplitude, width and relaxation time [4,56,57]. Since PA signal amplitude primarily depicts the optical absorption of the sample, which is wavelength dependent, different tissue chromophores such as haemoglobin, lipid, proteins, water, melanin (optical absorption spectra illustrated in Figure 5) can be probed by varying the excitation wavelength [58,59]. This is the fundamental principle of PA spectroscopy.

For example, oxygenated haemoglobin and deoxygenated haemoglobin is the signature for normal and malignant tissues and it has different optical absorption. Therefore, by tuning the wavelength one can differentiate these two types of tissues by PA spectroscopy [41,60].

### 3.3. PA Signal Analysis

In addition to amplitude, frequency domain features are also explored for extracting vital information such as shape/size/orientation of microstructures in tissue, acoustic scattering property and different biophysical properties of the sample [61,62]. It is directly related to the ultrasonic scattering and PA signal generation principle. The PA signal analysis resembles quantitative ultrasound (QUS) which is also a signal analysis-based technique for biological tissue characterisation. The PA signal analysis can be categorised into three major types, based on the underlying analysing techniques. This includes frequency spectra analysis, cepstral analysis and envelop statistics [63].

#### 3.3.1. Frequency Spectrum Analysis

The frequency spectrum analysis technique relies on the frequency components present in the PA time domain signal. The frequency components of the PA signal are primarily contributed by a different absorber present in biological tissue. The size, shape, attribution, and mechanobiological property dictate the characteristics of the PA frequency spectra. Eventually, the size and shape of the absorber can be determined from the PA power spectrum which is performed by first normalizing the PA power spectra by a reference spectrum.

(18) $$PS_{norm}\left(f\right)=\frac{1}{L}\sum_{l=1}^{L}log_{10}{\left(\frac{PS_{sample}{\left(f\right)}_{l}}{PS_{ref}\left(f\right)}\right)}^{2}$$

where, *PS_sample_(f)_l_* represents power spectrum of *l*th PA signal, *PS_ref_(f)* represents power spectrum obtained from reference.

In contrary to US, that uses transducer’s pulse-echo response for normalization, PA frequency spectrum utilises a reference spectrum from a gold-coated glass slide for spectrum normalization. The main reason is PA frequency spectra is wide band and also relies on single-way propagation. Subsequently, different spectral parameters such as slope and intercept are obtained by fitting the normalised PA spectra with a straight line within the transducer bandwidth (−6 dB),

(19) $$PS_{fit}\left(f\right)=SS\times f+Y_{int}$$

where, *SS* denotes the spectral slope and *Y_int_* denotes y-intercept of the regression line.

The midband fit can be obtained by spectral magnitude at the centre of the bandwidth utilised for analysis. The midband fit delineates information about concentration of the absorber, whereas the slope depicts the size of the absorber that serves as a fingerprint of many diseases, including cancer [62,64].

#### 3.3.2. Cepstral Analysis

The Cepstral analysis technique primarily relies on frequency content of the PA signal to determine the distance between two adjacent absorbers within densely populated absorbers, which is nearly impossible to differentiate with conventional ultrasound imaging. Since the cepstrum is highly sensitive towards repeated patterns, it is very effective to obtain periodic structures. In a recent study, this analysis has been employed to study chronic liver diseases, relying upon the dominant amplitude in the cepstrum. The cepstrum can be calculated from the time domain PA signal by applying the following equation:

(20) $$c\left(t\right)=F^{-1\;}\left\{log{\left|F\left(p\left(t\right)\right)\right|}^{2}\right\}$$

where, where *F* and *F*^−1^ represent the Fourier transform and the inverse Fourier transform of the PA time domain signal, respectively. The physical position of the absorbers can be readily determined with the temporal position of cepstral peaks and sound speed of the transmission medium. Then, the efficiency of the technique could be eventually improved by applying wavelet analysis which provides improved identification of recurring patterns in the cepstral harmonics.

The other approach of PA signal analysis was performed by Mahato et.al, in which different spectral parameters such as mean, median, standard deviation, total spectral energy etc. of magnitude spectra were used for tissue characterisation and classification [65,66,67]. These spectral features were correlated with changes in different biophysical property (protein concentration, binding and unbinding of protein) of the sample [11,66].

In addition, the mechanobiological property of the sample can be obtained from the PA frequency spectrum. The dominant frequency (frequency with maximum spectral magnitude) and mean frequency (mean of the frequency spectrum within a specified bandwidth) indicate the mechanobiological property of the sample.

#### 3.3.3. Envelope Statistics

The envelope statistic technique is based on the comparative statistical distribution analysis of the probability density function of the PA time domain signal envelope. The distribution parameters, e.g., fitting parameters, are mainly correlated to different biophysical parameters (size, temporal distribution, density) of the absorber. Subsequently, by fitting the probability density function to the PA signal envelope histogram, different diseases such as cancer and myocardial infraction were studied. In addition to the PA signal source, the underlying absorbing structures can also be revealed using this technique. Different probability distribution functions (pdf) (e.g., Rayleigh, Nakagami, Generalized gamma) are applied to the PA signal envelop histogram to analyse different fit parameters (e.g., *σ*, *m*, *c*, *v* and *a*), as illustrated in Equations (21)–(23), respectively. Among these pdfs, Rayleigh distribution is a one-parameter pdf. This has been widely used in detailing US backscattered signal envelop statistics for densely populated random scatterers. Equation (21) represents the Rayleigh pdf.

(21) $$R\;\left(A\right)=\frac{A}{\sigma^{2}}\;e^{-\frac{A^{2}}{\sigma }}$$

where, *σ* is the scale parameter. Subsequently, Equation (22) illustrate Nakagami pdf which is a double-parameter pdf that can detail about wide range of envelope statistics.

(22) $$N\;\left(A\right)=\frac{2m^{m}A^{2m-1}}{\Gamma \left(m\right)\;\Omega^{m}}\;e^{-\frac{m}{\Omega r^{2}}}U\left(A\right)$$

where, Γ is the gamma function, Ω and m represent the scale and shape parameter, respectively. Then, the generalised gamma pdf can be elucidated as Equation (23)

(23) $$G\;\left(A\right)=\frac{CA^{c\upsilon -1}}{a^{cv}\Gamma \left(\upsilon \right)}\;e^{-\frac{A}{a}c}$$

where, *a* is the gamma parameter, *c* and *υ* are shape parameters. As the generalised gamma pdf is a three-parameter pdf, it has the ability to provide details about the US signal statistics in a better way, compared to other pdfs [63].

## 4. Biomedical Application of PA Technique

### 4.1. PA Imaging Applications

Around the globe, researchers have worked on PA technique to take it from the laboratory bench top into hospitals. In the last decade, the PA technique has been applied to various biomedical applications, mainly for different disease diagnosis through imaging or signal analysis. Some of the major biomedical applications of PA technique are detailed as follows.

#### 4.1.1. Brain Imaging

Brain imaging in the present scenario is an essential requirement for disease diagnosis, as well as for studying the structural and functional operation of the brain. However, there are imaging modalities, especially with MRI, which is often used by clinicians, delineating certain disadvantages, such as exposure to a strong magnetic field and the cost. On the other hand, PAT can provide a cost-effective, non-ionic, real-time, high-resolution brain imaging technique [68,69,70,71,72]. It is capable of providing morphological as well as function information. A pioneer study performed by Dr. Wang and his group on a mouse brain illustrated a clear view of a lesion with intact skull. The study reveals a certain resemblance between the PAT image and the open skull image [73]. Later, this study was extended to observe tumour angiogenesis. This proves the capability of PAT for morphological brain imaging which will definitely be useful for brain tumour detection [72,74]. In another study conducted by the same research group, the researchers elucidated functional information, i.e., oxygen saturation in the mouse brain [68]. The brain activity due to whisker movements of the mouse were also studied by PAT. The abovementioned studies clearly illustrate that PAT has the strong potential to become a regular imaging tool for brain imaging. In addition to PAT, PAM is also employed to study the brain, and illustrated very promising results in terms of high resolution and ultrafast imaging [71,75,76,77,78].

#### 4.1.2. Arthritis Detection

Nowadays, arthritis is a major cause of disability around the globe. As PA imaging provides a high spatial resolution and deep penetration depth, it can be used for detection of arthritis as well. Xueding Wang et al., have studied joint structure of small animals using PAM. Further, they also imaged the human peripheral joint by PAT [79]. In this study, they found that optical absorption contrast of PA imaging provides the information about the tissue at the joint region better than the mechanical contrast of pulsed echo ultrasound imaging. The PAT images were compared with the gold standard technique, i.e., histopathology, that illustrated a very high correlation. PA combines with other imaging techniques (ultrasound) for image co-registration that provides structural as well as functional information [80,81,82]. Recently, light-emitting diodes (LEDs) are utilised as a sample excitation source instead of pulsed lasers, which helps to scale down the PA imaging apparatus size drastically. These LED-based PA imaging systems are employed for arthritis diagnosis, which demonstrate a comparable result to pulsed laser [83].

#### 4.1.3. Arterial Plaque Detection

**Figure 6:**
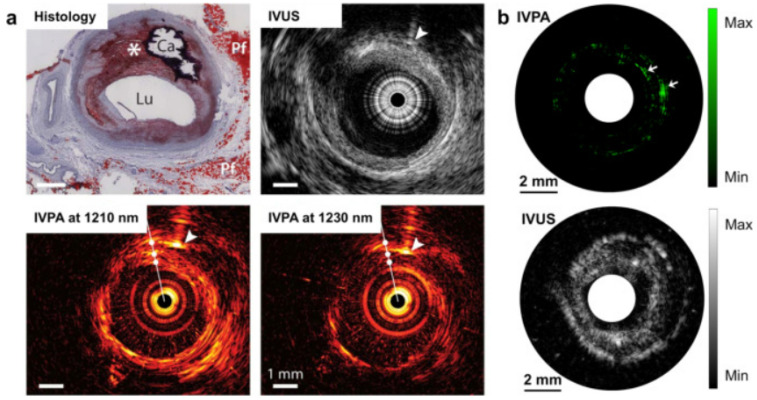
PA image of human artery in which dotted circle denotes the lipid-rich plaque [89]. (**a**) Intervascular photoacoustic (IVPA) and intervascular ultrasound (IVUA) image of an advanced atherosclerotic plaque in human (optical window 1.2 µm); (**b**) IVPA and IVUS imaging of excised human femoral artery (optical window 1.2 µm).

Heart diseases are very common nowadays. The major cause of several heart diseases is deposition of lipid-rich plaque in the arteries, which have a tendency to rupture [84,85]. These types of plaques may cause occlusive thrombus which may lead to heart attack or stroke [86]. By using miniature intravascular sideways-looking probe, the coronary artery wall can be imaged, which helps to detect the plaque. From the studies, it has been observed that lipid has an affinity towards 1210 nm light. Therefore, PA imaging could be helpful for lipid-rich plaque detection [87,88]. Although ultrasound imaging provides information about plaque, PA imaging provides more specific and quantitative information, as it provides better contrast than the ultrasound images. A study carried out by Allen et al. on excised human aorta exhibited that PAT provide a distinct contrast for lipid-rich plaque [87]. In an interesting study by Hui et. al, quantised vibration of a chemical bonding was used as the key parameter to identify lipid-rich plaque, as shown in Figure 6. The vibration-based PA imaging of atherosclerotic plaques illustrated promising results, as it correlated very well with standard histopathology [89]. Not only lipid deposition, but other plaque-forming causes such as calcium deposition, macrophage content, fibrous material can also be detected by PA imaging, as calcium, macrophage, fibrous all have a distinct optical absorption [90,91,92].

#### 4.1.4. Haematological Diseases

One of the applications of PA technique is haematological disease diagnosis. Since haemoglobin (chromophore present in red blood cells (RBCs)) has a strong affinity towards visible range wavelength [24], PA imaging has been explored for different disease diagnosis such as tumour angiogenesis, clot detection and differentiating acute and chronic clots in a phantom study, RBC aggregation, etc. [93,94,95,96,97,98,99,100]. Tumour angiogenesis is the fingerprint for malignant tissue detection, which is detailed in the later section. In this section, specifically haematological diseases are discussed.

Typical haematological diseases such as ischemia, myocardial infarction, deep vein thrombosis, stroke, etc., mainly arise due to blockage of blood vessels by clots or other obstacles such as lipid-rich plaque, macrophages, etc. [101]. Therefore, detection and analysis of blood clots is very important in terms of early diagnosis of these diseases [102]. Since haemoglobin strongly absorbs visible light, it will provide high contrast for a clot in the PA images. Based on this hypothesis, Emelianov and his group applied PA imaging to study aging of a blood clot in tissue phantom [93]. PA imaging of an acute and chronic clot was performed in tissue phantom. This clearly indicates that an acute and chronic clot exhibits distinct changes in contrast which can be utilised for discrimination of clots. The other application of the PA technique was explored by Kolios and his group in terms of PA spectrum analysis. They have studied RBC aggregation using simulation, as well as experimental studies through high frequency PA spectrum analysis [8,103]. The same group has also extended their work to observe changes in PA frequency spectrum due to oxygen saturation in pulsatile blood flow in an in vitro study. Not only RBC aggregation, but probing of single RBC morphology was also performed by the same group [104]. This proves the potential of the PA technique for haematological disease diagnosis.

#### 4.1.5. Cancer Diagnosis

Cancer is the deadliest disease that has taken millions of lives around the globe. In order to control the mortality rate, early-stage diagnosis as well as continuous monitoring is highly essential. Therefore, non-invasive and non-ionising PA imaging would be a promising option. The features of PA imaging have attracted the attention of many research groups worldwide. Since PA imaging relies on optical absorption of endogenous chromophores such as haemoglobin and melanin, malignant tissues exhibit very high contrast compared to normal tissues [1,60]. In one of the studies performed by Yang et al., growth of a melanoma tumour was observed. This illustrated very high contrast between the melanoma tumour and the surrounding tissues. Spectroscopic PA imaging of the skin melanoma tumour was performed with 584 nm and 764 nm wavelength that provided very high-resolution image of tumour vasculatures [105]. PA imaging has been taken to preclinical trials by Manohar and his group [32,106]. A photoacoustic mammogram has been developed and applied on human subjects to detect malignant breast tumours, Since vasculatures of malignant tissues are denser and abrupt compared to normal, it exhibits high contrast compared to normal tissues. In comparison to an X-ray mammogram or ultrasound, PAT exhibits more contrast. The same study also detailed the differentiation of a cyst from a malignant tumour [106].

**Figure 7:**
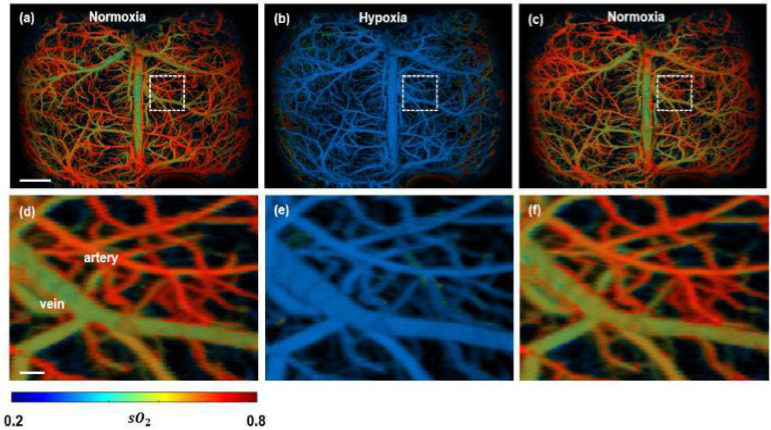
Functional PAM imaging of mouse brain hemodynamics under hypoxia. (**a**–**c**) sO_2_ images of the whole mouse cortex following the cycle of normoxia → hypoxia → normoxia. (**d**–**f**) Zoomed images of the dashed box part in (**a**–**c**) [77].

In contrast to relying on tumour vasculature, PA imaging has been utilised to extract functional information of the tissue, i.e., oxygen saturation in blood (hypoxia) is monitored [107,108,109]. An obtained spectroscopic PA image is illustrated in Figure 7. The malignant tumour inside a mouse brain can be observed with high contrast, which indicates the hypoxic region very prominently.

Another primary reason of cancer related death is due to metastatic spread of the primary tumour. Identification of circulating tumour cells (CTC) would be very useful to slash the mortality rate [110]. PA imaging is applied to detection of CTC in blood stream. In vivo detection of CTC in the blood stream provides a manyfold increase in sensitivity compared to existing in vitro techniques [97,111,112]. Subsequently, use of exogenous contrast agents increases the sensitivity and specificity.

In order to enhance the imaging capability of PAI, different exogenous contrast agents are used. Different near infrared (NIR) absorbing dyes such as indocyanin green, Alexa flour750 and IRDye800CW are being commonly used as contrast-enhancing agents [113,114]. In addition to these NIR dyes, gold nanoparticles (GNPs) are very popular as contrast-enhancing agents. Different types of GNP such as nano rod, nano shell, nano cages and nano beacons are used for PAI imaging [114,115,116,117]. The major applications of these NP are for targeted imaging applications. Besides PA imaging, PA spectroscopy is also explored for cancer diagnosis. It has been applied to ovarian, breast and solitary thyroid tissues for differentiating normal from malignant tissues [65,118].

Some more details are illustrated in Table 1.

### 4.2. PA Signal Analysis Applications

#### 4.2.1. Tumor Diagnosis

From the previous section, we have observed that PA imaging is widely applied for cancer diagnosis. In addition to PA imaging, PA signal analysis is also employed for studying and differentiating tumors based on different biophysical parameters such as scatterer size, concentration, mechanobiological property, etc. [10,104,120,121]. Since RBCs consist of huge optical absorption cross-sections, blood vessels filled with RBCs are considered as the primary source of PA signals from tumors [60,100]. The PA time domain signal obtained from different tumors is analyzed using the PA signal analysis technique illustrated in Section 3.3 which provided different spectral parameters correlating to the physical structure of the tissue sample.

One such study revealed that in the case of closely filled, non-differentiable PA absorbers, the change in absorber size and density can be computed using the generalized gamma envelope statistic fit parameter *a* [63]. This approach could be effectually used for monitoring biophysical changes in the vascular structure around the tumor. In another study by Wang et. al, PA spectral slope, mid-band fit and intercept was used to characterize prostate adenocarcinoma tumor in a murine model. They reported significant decrease in PA mid-band fit and intercept in tumor compared to normal tissue, whereas the PA slope enhances notably [64]. Recently, Guan et al. employed PA spectral analysis for determining the Gleason grade of prostate cancer. The authors used a 266 nm wavelength to probe the cell nuclei which provided significant architecture heterogeneity in normal, early stage and late-stage cancer [119]. Further, multiple wavelength PA signal processing was also explored for differentiating normal, benign and malignant prostate [122]. Primarily, freshly excised human prostate was used to acquire PA time domain signal with 760 nm and 800 nm. They found a prominent difference in three spectral parameters, midband fit, intercept and slope (*p* < 0.01), among malignant, benign and normal prostate tissue. In a current study, wavelet transform-based PA spectral analysis was used for grading the prostate tumor based on Gleason scores (GSs). This study utilized 1210 nm and 1310 nm wavelengths to obtain PA frequency spectra, which delineated a high slope for high GSs [123]. In a current study, machine learning-assisted PA spectroscopy was utilized to identify prostate cancer. The authors employed unsupervised hierarchical clustering and supervised classification for prostate malignancy diagnosis. This study reveals strong correlation between the PA power spectra and biochemical components in case of malignant tissue compared to normal tissue which leads to momentous classification efficiency (82%) [13]. Apart from prostate cancer, PA signal analysis is also employed for detection of breast malignancy. Biswas et al. utilized time-frequency-based advanced signal processing tool Wigner–Ville distribution (WVD)-based PA frequency spectra to differentiate normal and malignant breast tissue, relying upon the mechanobiological property of these two tissue types [124]. This study reported two dominant frequency components in case of malignant breast tissue, whereas the normal breast tissue illustrated a single frequency peak. In another study, they employed empirical wavelet transform (EWT)-based PA frequency spectra analysis for differentiating various biopsy excised breast tissues, e.g., normal, benign and malignant tissues. Authors reported very high accuracy for different breast tissue types and high correlation with histopathology [125]. In addition to pulsed PA, there are several literatures on continuous PA frequency spectrum analysis. Gorey et al. developed a continuous PA system, which was employed for breast carcinoma diagnosis. The PA time domain signal was obtained from biopsy excised breast tissue in continuous mode. Further spectral energy was calculated from PA frequency spectra of normal and malignant tissues, which illustrated that malignant tissues elucidate higher spectral energy compared to normal tissues [126]. Choi et al. reported wavelength modulated differential photoacoustic spectroscopy (WM-DPAS) for early-stage cancer diagnosis through change in total hemoglobin concentration and oxygenation levels (StO_2_). The authors reported a 162.84%/% change in amplitude of the PA signal due to a 1% change in StO_2_ [127].

#### 4.2.2. Single Cell Characterization

Tissue microstructure is considered as a fingerprint to identify different diseases, including cancer [121]. Since PA frequency spectral analysis is capable of detecting microstructure in tissue phantoms as well biological tissues, it has been widely explored by researchers worldwide in different applications. Xu et.al studied PA frequency spectral from a single microstructure of 300 µm and dispersed microsphere of different diameter (100, 200, 300, 400 and 500 μm) in a simulation and experimental study. The authors theoretically validated the PA frequency spectrum and the analytical solution to the PA frequency spectrum was validated [121]. In another study, the relationship between an absorber’s characteristics and different PA frequency spectral parameters (midband fit, slope, intercept) was explored [128]. Authors reported spectral slope decrease promptly with the decrease in diameter of the absorber whereas it slightly decreases when the concentration of the absorber decreases. The midband fit and intercept increase with both increase in diameter and concentration of the absorber. Later, PA spectral analysis was explored for studying blood cells. Hysi et al. employed PA frequency spectral analysis for studying blood cell aggregation. They reported enhancement in mid-band fit due to increase in hematocrit concentration as well as aggregation, whereas the spectral slope decreases with increase in aggregate size [129]. Bok et al. studied the relationship between RBC aggregation and oxygen saturation during pulsatile blood flow. The obtained result clearly elucidated the aggregation of RBC directly influences the absorber size and absorption coefficient [130]. Subsequently, the effect of an ultra-wide band optical-ultrasound detector on the PA frequency spectrum was explored by Feng et. Al. In this study, first, a microsphere was studied with a micro ring resonator (optical) and needle hydrophone (piezoelectric). Among these two detectors, the ultra-wide band micro ring detector illustrated higher difference in the spectral slope due to change in size of the absorber. Later, applied to fresh (7.82 μm) and aged RBC (5.68 μm), the micro ring detector was able to differentiate among these two types of RBCs through significant difference in the spectral slope [131]. There are other studies which have probed change in the RBC shape using PA frequency spectral analysis in simulation as well as through experimental studies [100,104]. In addition, some reports also explored PA frequency spectra as a tool to differentiate melanoma from RBCs [132], normal and malaria-infected RBCs [133] tumor cells and RBCs. An interesting study was performed by Fadhel et al., in which RBC death was probed with multispectral PA frequency spectra. RBC death was probed by computing the quantity of the by-product (methemoglobin) produced during RBCs depth with multispectral PA frequency spectra. By using a spectral unmixing approach, the authors detected a 7% enhancement in methemoglobin concentration [134].

#### 4.2.3. Miscellaneous Applications

PA frequency spectral analysis is employed to other different applications. One such application is atherosclerotic plaques in the coronary artery, which is also a life-threatening condition. Hence, non-invasive detection is evident. PA frequency spectral analysis alongside PA imaging is also employed for plaque detection. Daeichin et al. combined spectroscopic PA imaging with PA frequency spectrum analysis for studying human coronary arteries in an ex-vivo study. In this study, a wide band ultrasonic detector (1–35 MHz) was used for PA signal acquisition and a laser wavelength 1125 to 1275 nm was used for sample excitation. The authors reported >80% PA spectral energy was concentrated below 8 MHz frequency band in the case of a lipid-rich coronary plaque [135]. In another study, Cao et al. explored PA signal analysis to differentiate and also estimate the lipid structure present in the artery wall. The authors used the k-means clustering method to classify PA frequency spectral response. This study reported 98.4% accuracy in differentiating lipid in the phantom [136]. PA frequency spectral analysis is also utilized for characterizing bone microstructures. Feng et al. reported that the spectral slope increases when there is a decrease in thickness of the trabecular in a simulation study. The same study also reports good correlation with simulation and experimental study [137]. Recently, Xie et al. employed wavelet transform-assisted PA spectral analysis for assessment of bone mineral density (BMD) and structure of bone. The result delineated bone with lower BMD and thinner trabecular thickness had high frequency components. The midband-fit and slope can differentiate normal and osteoporotic bone with high sensitivity [14]. The same group very recently presented a physiochemical spectrogram from bone with multi-wavelength PA sensing. The spectral slope is used as the key parameter to study physical and chemical properties of bone [138]. PA spectral analysis is employed for distinguishing normal and pneumonia-affected lungs, by Biswas et al. They reported higher dominant frequency in pneumonia-affected lungs compared to normal lung tissue [139]. The same authors also applied PA frequency spectral analysis to differentiate blood and a blood clot. The blood clot illustrated approximately two times higher dominant frequency compared to blood [10]. Another application of PA frequency spectral analysis is blood glucose detection. Long et al. utilized the PA time-frequency spectrum to obtain Teager–Kaiser main energy, which was correlated to glucose concentration [140].

## 5. Conclusions

The photoacoustic technique is a non-invasive, non-ionizing technique that uses light for sample excitation and ultrasonic detection. Due to this unique feature, PA is explored for different biomedical applications. PA imaging and PA frequency spectral analysis has been widely explored by the scientific community worldwide. The PA technique has been utilized to study different diseases, including cancer. Despite such huge number of studies on PA technique, it is still mostly a lab-scale technique. It is yet to become a regular clinical tool. There should more studies along this line in the near future. The primary challenge of the PA technique is the pulsed laser which is used for sample excitation. This not only makes the system costly but also demands a larger operating area, as well as high operational energy. Therefore, alternative to pulsed lasers, researchers are exploring different alternatives such as laser diodes and LEDs as PA excitation sources. In addition, the depth of penetration of the PA technique is also low compared to other conventional techniques such as US, MRI, etc. Hence, researchers are using external contrast agents and also endoscopic techniques for deep tissue PA imaging. In addition, PA imaging can be coupled with PA signal analysis which would provide more critical information about the sample. This would complement the limitations of the exciting PA technique.

## Figures and Tables

**Table 1:** Different Photoacoustic Technologies and its applications.

PA Imaging Technology	Application Area	Reported Laser Wavelength	Reference
PAT	Brain	584- and 600-nm	[68]
PAM	Brain Imaging	570 and 578 nm	[69]
Photoacoustic computed tomography	Human Brain	1064 nm and 694 nm	[71]
OR-PAM	Awake mouse brain imaging	532 nm	[72]
Photoacoustic computed tomography	Development and treatment of rheumatoid arthritis	780 nm	[80]
PAT	Human Musculoskeletal Imaging and Inflammatory Arthritis detection	720-nm	[82]
LED based PAT	Joint Inflammation	850-nm wavelength	[83]
Vibration-based PA imaging	Lipid-laden plaques	400 nm to 1.1 μm	[89]
Photoacoustic spectral analysis	Prostate cancer	266 nm	[119]
PAT	Breast tumour imaging	1064 nm	[32]
Photoacoustic and ultrasound (PAUS) assembly	Porcine Pancreatic Cancer imaging	680–950 nm	[116]

## Data Availability

Not applicable.
